# Harnessing Oncolytic Viruses for Targeted Therapy in Triple-Negative Breast Cancer

**DOI:** 10.7150/ijms.105683

**Published:** 2025-04-13

**Authors:** Shasha Tang, Liyun Yong, Yong Cui, Haibin Li, Evelyne Bischof, Fengfeng Cai

**Affiliations:** 1 Department of Breast Surgery, Tongji Hospital, School of Medicine, Tongji University, 389 Xincun Rd, Shanghai 200065, China.; 2 Department of General Surgery, People's Hospital of Otog Qianqi, Sharita Tara East Street, Aolezhaoqi Town, Otog Qianqi 016200, China.; 3 Department of Medical Oncology, Renji Hospital, School of Medicine, Shanghai Jiao Tong University, Shanghai, China.; 4 Shanghai University of Medicine and Health Sciences, Shanghai, China.

**Keywords:** Triple-negative breast cancer, oncolytic virus, immunotherapy, targeted therapy, combined therapy

## Abstract

Breast cancer is the most prevalent malignant tumor among women, with triple-negative breast cancer (TNBC) being one of the most aggressive forms due to its high invasiveness and metastatic potential. Traditional treatments such as endocrine therapy and anti-HER2-targeted therapy are largely ineffective for TNBC, and while chemotherapy shows some promise, resistance remains a significant hurdle. Recently, there has been increasing interest in biological therapies, especially oncolytic viruses (OVs). OVs promote anti-tumor effects by selectively killing tumor cells and stimulating immune responses, and have achieved notable breakthroughs in breast cancer treatment. OVs have demonstrated effectiveness comparable to surgery, radiotherapy, or chemotherapy in selected cancers, but data are sparse in the context of TNBC. This review provides an overview of recent progress in the application of OVs as a tool for precision TNBC treatment.

## Introduction

Breast cancer (BC) presents itself as a multifaceted and diverse malignancy, posing a considerable burden on global health. It stands as the most prevalent cancer and the primary contributor to female cancer-related deaths worldwide 1-3. Understanding the diverse forms of BC is essential for accurate diagnosis, prognosis, and treatment planning 4. While current treatments have significantly improved outcomes, they still have limitations, prompting the need for new therapeutic approaches 5. BC arises from the uncontrolled growth of ductal or lobular cells in the breast tissue. The disease's numerous molecular subtypes and clinical presentations influence its progression and response to treatment 6, 7. BC presents a formidable challenge in global healthcare, necessitating the advancement of innovative therapeutic strategies to enhance patient outcomes.

BC is characterized by high heterogeneity. Based on the expression of immunohistochemical markers such as progesterone receptor (PR), estrogen receptor (ER), human epidermal growth factor receptor-2 (HER2), and Ki-67, breast cancer can be classified into four subtypes: Luminal A, Luminal B, HER-2 positive (HER2+), and triple-negative breast cancer (TNBC), where the latter is negative for all three markers (ER, PR, HER2). TNBC is notably aggressive, prone to early recurrence and metastasis, and has a poor prognosis, accounting for about 15-20% of all breast cancer cases 8. TNBC patients do not benefit from endocrine therapy or HER2 targeted therapy, with chemotherapy remaining the main treatment option. However, its effectiveness is very limited, and the overall survival time for advanced TNBC patients is only 13-18 months 9. While some patients may benefit from targeted poly ADP-ribose polymerase (PARP) therapy, new targeted treatments for TNBC are still in the early stages 10. Further exploration of precision treatment strategies for TNBC to improve patient outcomes and survival is a future research direction.

**Figure 1:**
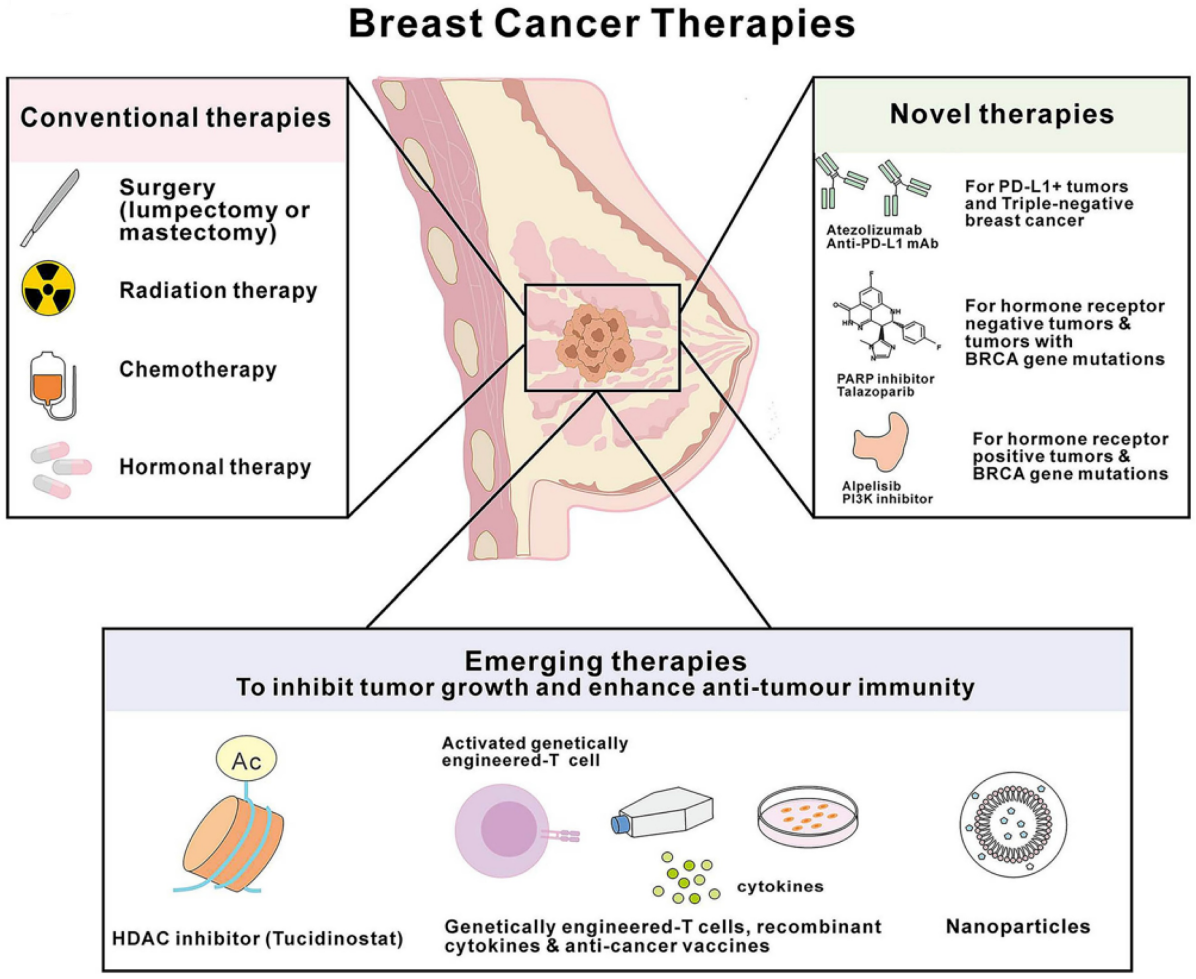
Schematic representation of current treatment modalities for breast cancer.

TNBC is considered the most immunogenic subtype, with higher levels of tumor-infiltrating lymphocytes (TILs), programmed cell death-ligand 1 (PD-L1) expression, and tumor mutation burden (TMB) compared to other subtypes. This suggests that the tumor microenvironment (TME) of TNBC has strong immune activity, providing a basis for the application and promotion of immunotherapy in TNBC 11. Current breast cancer immunotherapy research is mainly focused on TNBC, and immunotherapy is transforming the current treatment model of TNBC (**Figure 1**).

Tumor immunotherapy aims to enhance the body's immune system, restore the body's anti-tumor immune response, and thereby control and eliminate tumors. TNBC immunotherapy can be roughly divided into four categories: immune checkpoint inhibitors (ICIs), adoptive cell transfer therapy (ACT), cancer vaccines (CVs), and oncolytic viruses (OVs). Immune checkpoints are inhibitory molecules present on the surface of cells, primarily used to regulate the function of T cells to maintain the balance of the immune system. PD-1, PD-L1, and CTLA-4 are currently the most studied immune checkpoint inhibitors 12-14. To date, various ICIs have been approved by the U.S. Food and Drug Administration, including PD-1 inhibitors Pembrolizumab and Cemiplimab, PD-L1 inhibitors Atezolizumab and Avelumab, as well as CTLA-4 inhibitors Tremelimumab and Ipilimumab 12. Numerous clinical trials have explored the efficacy of PD-1, PD-L1, and CTLA-4 inhibitors in TNBC, including series studies such as KEYNOTE, IMpassion, and BreastImmune03^13^, ^15^, ^16^. Although many clinical trials indicate that ICIs combined with chemotherapy can bring survival benefits to TNBC patients, some patients still respond poorly to this treatment strategy.

ACT therapy mainly includes CAR-T, TILs, and TCR-T therapies, all aiming to enhance the anti-tumor ability of T cells. Currently, at least 12 early clinical studies are evaluating the efficacy and safety of CAR-T therapy in TNBC, mainly targeting ROR1, TEM8, and EGFR 17. TCR-T therapy has been developed for over 20 years, and multiple preclinical studies have shown that TCR-T therapy targeting tumor-specific antigens such as MAGE and GP100 has certain efficacy in melanoma, colorectal cancer, and synovial sarcoma 10, but clinical trials of TCR-T for TNBC treatment are relatively few. In addition, cancer vaccines are currently a major focus in the field of tumor immunotherapy. The basic principle is to use various forms such as peptides, nucleic acids, and cells to introduce tumor-associated antigens into the patient's body to stimulate the immune response and thus achieve the goal of preventing or treating tumors. TNBC vaccines currently in clinical research include NeuVax, Adagloxad Simolenin vaccine, and α-lactalbumin vaccine 18, among which only a few vaccines have entered phase III clinical trials 19.

Although the above three immunotherapies have improved the prognosis of many TNBC patients, only a portion of patients benefit due to lack of targets, high toxicity, and treatment resistance. Therefore, developing new precision immunotherapy methods is expected to provide new ideas for TNBC treatment, and oncolytic virus immunotherapy is one such approach.

While several reviews have explored the potential of oncolytic virotherapy for triple-negative breast cancer (TNBC), this review distinguishes itself by providing a comprehensive analysis of the latest advancements in engineered oncolytic viruses, particularly focusing ontheir ability to overcome resistance mechanisms in TNBC. Unlike previous reviews, we emphasize the integration of oncolytic viruses with emerging immunotherapies, such as immune checkpoint inhibitors and CAR-T cell therapy, and discuss the potential of combination therapies to enhance therapeutic outcomes. Additionally, we provide an in-depth analysis of ongoing clinical trials and highlight the challenges of translating preclinical findings into clinical practice, offering a forward-looking perspective on the future of oncolytic virotherapy in TNBC treatment.

### Oncolytic viruses

Oncolytic virus therapy has become a groundbreaking and promising approach in cancer treatment 20. These viruses, which can be either genetically engineered or naturally occurring, possess the unique ability to selectively destroy cancer cells while leaving normal tissues unharmed 21. This fundamental difference distinguishes oncolytic virus therapy from traditional gene therapy, where viruses mainly serve as vectors to deliver therapeutic genes. In oncolytic virus therapy, the virus itself acts as a therapeutic agent, directly attacking and eliminating cancer cells. The relationship between viruses and tumor cells was first observed in the early 1800s when patients showed tumor reduction after viral infections 22. To date, four oncolytic viruses have been approved globally for cancer treatment. The first approval came in 2004 for Rigvir, an RNA virus derived from the native ECHO-7 strain of picornavirus, for melanoma treatment in Latvia. Following this, in 2005, China approved H101, a genetically modified adenovirus, for use with cytotoxic chemotherapy to treat nasopharyngeal carcinoma. In 2015, the U.S. Food and Drug Administration approved Talimogene laherparepvec (T-VEC), an attenuated herpes simplex virus type 1 (HSV-1) encoding granulocyte-macrophage colony-stimulating factor (GM-CSF), for the local treatment of melanoma 23. In June 2021, Japan approved the oncolytic HSV drug Delytact (teserpaturev/G-47delta) for the treatment of glioblastoma, the first approval of an oncolytic virus in the world for the treatment of primary brain cancer or glioblastoma. The approval is based on the results of Dr. Todo's Phase 2 clinical trial in patients with glioblastoma 24, which demonstrated the safety and efficacy. This approval signifies a major milestone in the field of oncolytic virotherapy, as it expands the potential applications of oncolytic viruses beyond melanoma and other cancers to highly aggressive and difficult-to-treat tumors and opens new avenues for exploring the use of oncolytic viruses as a targeted, immunotherapeutic option in oncology, potentially transforming the landscape of cancer treatment in the future.

## Oncolytic viral mechanisms of action

**Figure 2:**
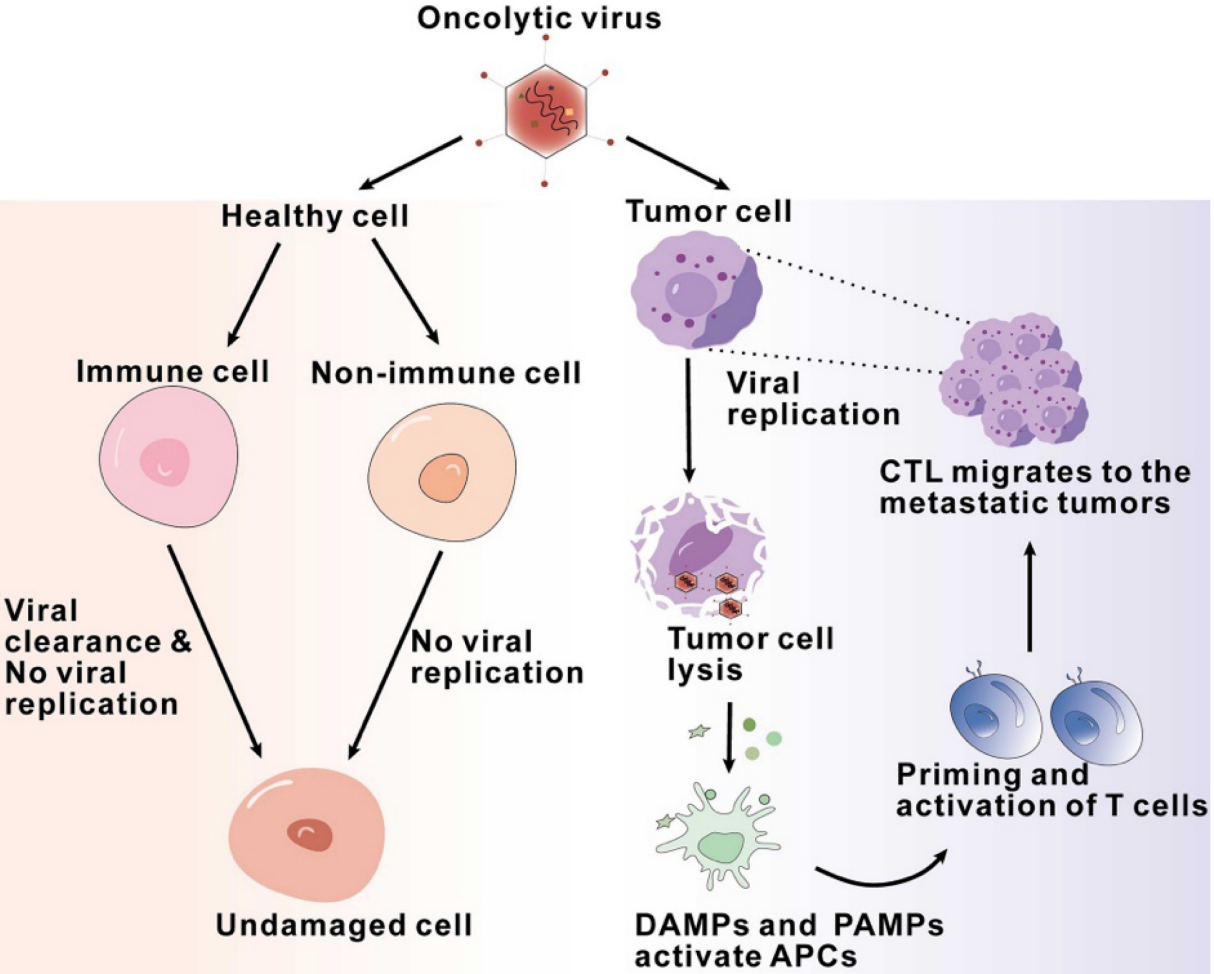
Flowchart of antitumor mechanisms of oncolytic viruses, illustrating the sequence of interactions and processes of oncolytic viruses exerting their effects within the tumor microenvironment.

Firstly, OVs have the ability to directly dissolve tumor cells. Normal host cells sense viral components and clear viruses by activating signaling pathways, while abnormal antiviral mechanisms in tumor cells allow the survival and replication of viruses 25, 26. Naturally occurring or genetically modified Ovs target defective antiviral pathways within tumor cells and selectively infect, replicate, and lyse cancer cells while leaving normal cells unharmed 27, 28. Tumor cells infected with oncolytic viruses lyse and release viral particles that spread to surrounding uninfected tumor cells, leading to the expansion of oncolytic activity 29. In addition, recent studies have found that tumor cells can release exosomes after being infected with oncolytic adenovirus (Oad), which can also enhance anti-tumor efficacy 30 (**Figure 2**).

Secondly, Ovs activate the innate immune system. After infection, viral components known as pathogen-associated molecular patterns (PAMPs), including viral capsids, DNA, RNA, and proteins, are exposed to the host immune system, activating an immune response 31. Furthermore, Ovs mediate immunogenic cell death (ICD), including immunogenic apoptosis, necrosis, and pyroptosis, which induce endoplasmic reticulum stress and lead to the release of damage-associated molecular patterns (DAMPs) such as ATP, high mobility group box 1 (HMGB1), heat shock proteins, calreticulin, and pro-inflammatory cytokines 30, 32, 33. These PAMPs and DAMPs are recognized by pattern recognition receptors (PRRs) on immune cells, such as stimulator of interferon genes (STING), Toll-like receptor (TLR) adaptor molecule 1, and TLR3. This recognition subsequently results in immune cells producing and secreting pro-inflammatory cytokines and chemokines, such as interferons (IFNs), interleukins (IL-1β, IL-6, IL-12), tumor necrosis factor-α (TNF-α), granulocyte-macrophage colony-stimulating factor (GM-CSF), and various chemokines. The production and release of these cytokines can establish an inflammatory tumor microenvironment, transforming immunogenically “cold” tumors into “hot” tumors 34. Firstly, the secreted chemokines can recruit initial responder cells, such as neutrophils and macrophages, to the infected area, thereby triggering an effective anti-tumor immune response 35, 36. The pattern recognition receptors on natural killer (NK) cells bind to pathogen-associated molecular patterns, leading to the activation of NK cells, which then migrate to the virus-infected area 36. Subsequently, the activated NK cells release granzyme and perforin and trigger the FAS-FASL signaling pathway, resulting in the killing of virus-infected cells^34^. Then, these NK cells secrete IFN-γ and TNF-α, which not only promote the activation of macrophages, dendritic cells, and T cells but also further enhance the production of immune response factors, including IFN, TNF-α, IL-12, IL-6, and chemokines, thereby strengthening the innate immune response^36^, ^37^.

Finally, Ovs activate antitumor adaptive immunity. During OV infection, the body primarily achieves antitumor adaptive immune responses through tumor-specific T cell responses. The antigen-specific activation of T cells depends on three signals provided by antigen-presenting cells (APCs): the antigen epitope presented by MHC molecules, the involvement of co-stimulatory molecules, and the action of cytokines. The lysis of tumor cells caused by Ovs results in the release of tumor-associated antigens (TAAs) and neoantigens. These antigens are processed by APCs to form antigen epitopes, which bind to MHC molecules and are presented on the surface of APCs. In the cytokine environment generated by the exposure of immune and tumor cells to Ovs, type I interferons (IFNs) enhance the expression of MHC molecules and co-stimulatory molecules on the surface of dendritic cells 38. Numerous studies have demonstrated that Ovs can induce the activation of MHC class I-related molecules and co-stimulatory molecules, thereby successfully activating antigen-specific T cell responses 38, 39. Cells infected by Ovs or mature APCs can produce various cytokines and chemokines, which aid in the recruitment and reactivation of T cells. Once activated, these antitumor CD8+ T cells and B cells promote tumor regression and eliminate newly implanted or distant tumors in an OV-independent manner 40.

## Oncolytic virus in breast cancer

Research on OVs for breast cancer has reached the clinical trials stage, demonstrating highly promising potential as a future cancer treatment due to their ability to selectively target tumor cells 41. In both preclinical and clinical trials, the focus has primarily been on four virus groups from the seven groups in the Baltimore classification: group I (double-stranded DNA viruses), group III (double-stranded RNA viruses), group IV (single-stranded RNA viruses - positive-sense), and group V (single-stranded RNA viruses - negative-sense). These include viruses that are naturally anti-neoplastic, those engineered for tumor-selective replication, and those genetically modified to activate the immune system 41, 42. At present, no OV is officially registered for breast cancer treatment.** Table 1** lists the OVs being tested in clinical trials for breast cancer, both as monotherapy and in combination therapies. Additionally, numerous preclinical trials are underway, exploring various viruses from these four groups.

Numerous preclinical trials are dedicated to discovering an effective OV for TNBC. While chemotherapy is the standard treatment for these patients, it is associated with severe side effects, frequent drug resistance, and a poor prognosis. The challenges in treating TNBC and its high heterogeneity have spurred many studies aimed at developing more efficient and safer treatment options 43, 44.

Adenoviruses are the most extensively studied OVs in breast cancer. Preclinical studies, including those with additional modifications (such as the insertion of antitumor and immune regulatory genes to enhance effects), have also targeted TNBC 45. One example is a recombinant type five adenovirus containing the IL-24 gene (CNHK600-IL24), which significantly suppressed tumor growth in a nude mice model and improved survival in a metastatic model 46. Another OV demonstrating high efficacy against MDA-MB-435 cancer cells is G47D, an oncolytic HSV (approved for Malignant Glioma), which exhibited strong cytotoxicity against human breast cancer cells *in vitro* and in tumor xenografts *in vivo* 46, 47. Additionally, the VG9-IL-24 recombinant Vaccinia virus showed promising effects against MDA-MB-231 TNBC cells, demonstrating efficiency in infecting and selectively killing breast cancer cells without strong cytotoxicity to normal cells in a xenograft mouse model 47. Ji et al utilized *in vitro* experiments and mouse breast cancer models to evaluate the molecular mechanisms by which ApoA1 regulates cholesterol efflux and inhibits cancer progression and metastasis. The researchers inserted the gene encoding ApoA1 into the adenovirus genome to construct a recombinant adenovirus (ADV-ApoA1). Subsequently, they assessed the efficacy of ADV-ApoA1 in inhibiting TNBC growth and metastasis in several mouse models, including orthotopic breast cancer, spontaneous breast cancer, and human breast cancer xenografts. Additionally, comprehensive safety evaluations of ADV-ApoA1 were conducted in Syrian hamsters and rhesus monkeys 48.

A recent case study examined a patient previously treated for metastatic triple-negative breast cancer (mTNBC). The study aimed to assess the safety and effectiveness of CHECKvacc, an oncolytic virus comprising CF33, a chimeric vaccinia poxvirus. Although the initial intratumoral administration showed no immediate response, subsequent treatment with T-Dxd resulted in significant tumor regression and a disease-free survival period of 10 months 49.

Likewise, research findings indicate that adenovirus carrying interleukin 12 (IL-12) not only efficiently inhibits tumor progression in the 4T1 syngeneic TNBC mouse model but also induces abscopal effects. These effects, a seldom-seen occurrence, involve the treatment of a tumor at one location resulting in the regression of metastatic tumors at remote sites. This phenomenon is mainly ascribed to the activation of immune cells and the suppression of tumor angiogenesis 50.

Although oncolytic viruses hold promise as a potential treatment avenue for breast cancer, it's becoming increasingly clear that relying solely on them may not suffice for comprehensive and effective treatment 51. Breast cancer is a multifaceted and diverse condition, often involving various mechanisms and pathways that can hinder the effectiveness of oncolytic viruses in isolation 43. Therefore, integrating oncolytic viruses with other therapeutic approaches, such as chemotherapy, immunotherapy, or targeted therapies, may present a more robust and comprehensive strategy for addressing breast cancer 52. These combined treatments have the potential to target multiple facets of the disease, bolster the immune response, and overcome resistance mechanisms, ultimately enhancing outcomes for breast cancer patients.

In 2017, phase I and II clinical studies were initiated to investigate the combination therapy of oncolytic virus (talimogene laherparepvec, T-VEC) with standard chemotherapy for early-stage triple-negative breast cancer (TNBC). T-VEC is a modified type of herpes simplex 1 virus, encoding granulocyte-macrophage colony-stimulating factor (GM-CSF). The chemotherapy regimen included anthracycline and taxane. The rationale for using T-VEC in combination with chemotherapy was the enhanced response of TNBC tumor lesions infiltrated by lymphocytes to such neoadjuvant therapy. Preclinical data also supported the synergistic effect of oncolytic virus and chemotherapy. However, progress in these clinical trials has been relatively slow. Based on known preclinical and clinical outcomes, it is speculated that the oncolytic and immune activation effects demonstrated by T-VEC in melanoma patients may enhance the responsiveness of TNBC to T-VEC during neoadjuvant chemotherapy. This concept has been validated through phase I clinical trials, confirming its safety and reliability 53.

In the KEYNOTE-522 clinical trial, the combination therapy of the PD-1 inhibitor pembrolizumab with chemotherapy showed a significant improvement in efficacy compared to chemotherapy alone. The probability of achieving pathological complete response was 64% with the combination therapy, while it was 51% with chemotherapy alone. The three-year event-free survival rate was 84.5% with combination therapy, compared to 76.8% with chemotherapy alone. Therefore, the Food and Drug Administration (FDA) approved the combination of pembrolizumab and chemotherapy. Although the efficacy of the above-mentioned treatment regimen is significant, it also entails considerations such as potential autoimmune toxicities. The addition of additional systemic immunotherapy drugs within this framework has indeed increased the probability of autoimmune toxicities. However, treatment regimens such as oncolytic viruses, which can be administered locally, can activate anti-tumor immunity without increasing the risk of systemic toxicities 54.

## Oncolytic virus in triple negative breast cancer

**Figure 3:**
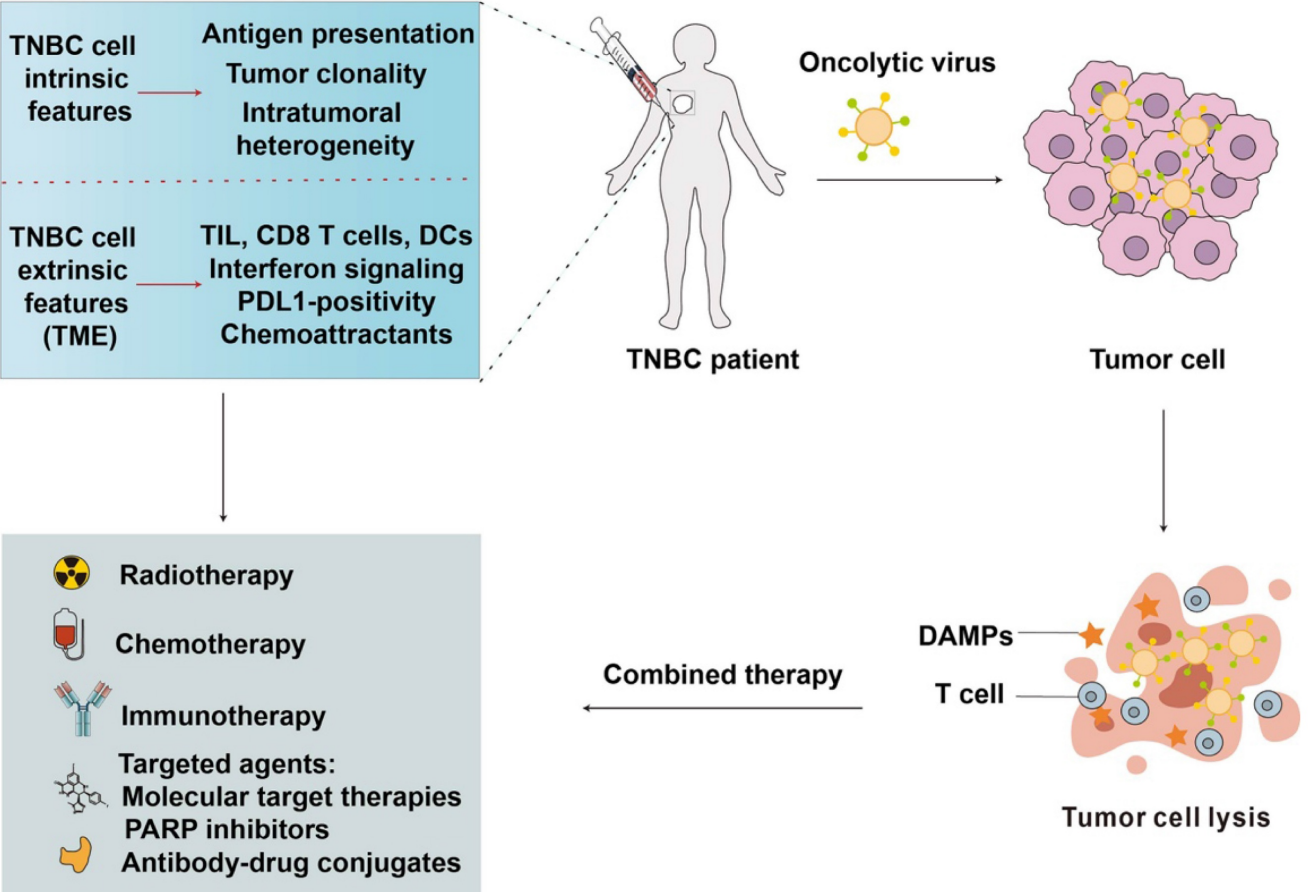
Integration of oncolytic viruses with other anticancer therapies for TNBC.

TNBC patients often have fewer treatment options and poorer prognoses, leading to a pressing need for innovative approaches. OVs offer a promising avenue for addressing these challenges, as they selectively infect and destroy cancer cells while sparing normal tissue 55. OVs can also stimulate the immune system, enhancing the anti-tumor response, which is particularly beneficial in TNBC's immunologically cold tumors. By targeting the tumor microenvironment and inducing immunogenic cell death, OVs may overcome the resistance often encountered in traditional TNBC treatments, offering a novel and potentially more effective therapeutic strategy (**Figure 3**).

Adenoviruses have shown great promise in the treatment of TNBC through various innovative approaches. Adenoviruses are often used in combination with other therapeutic methods to boost their effectiveness. For example, a phase II study is evaluating the effects of adenovirus-mediated herpes simplex virus thymidine kinase expression combined with valacyclovir, stereotactic radiotherapy, and pembrolizumab in patients with TNBC 56. The combination of CAR-T therapy with oncolytic adenoviruses has been explored to improve outcomes in TNBC 57. Li et al. evaluated the combination of CAR-T therapy and an oncolytic adenovirus expressing TNFβ receptor II-Fc in a TNBC model, which targets and inhibits TNFβ signaling, thereby decreasing immunosuppressive effects in the tumor microenvironment 58. The study found improved CAR-T cell migration and performance when mesothelin-targeted CAR-T-cell therapy was combined with an oncolytic virus expressing TGF-β 58.

Researchers have developed adenoviruses encoding specific proteins to target TNBC. For example, an adenovirus encoding the full-length human HER3 receptor (Ad-HER3) was generated as a potential cancer vaccine 59. This vaccine not only induced strong T-cell anti-tumor responses but also provided additional activity to eliminate tumors where HER3 signaling mediates aggressive behavior or acquired resistance to HER2-targeted therapy and TNBC. Oncolytic adenoviruses are frequently combined with immune checkpoint inhibitors (ICIs) to improve therapeutic outcomes. Since the FDA approval of T-VEC for melanoma, many oncolytic viruses, including adenoviruses, have demonstrated modest anti-tumor efficacies with tolerable toxicity profiles. Researchers are now combining genetically modified oncolytic viruses with ICIs to enhance their effectiveness 60. The therapeutic use of oncolytic adenoviruses involves overcoming several barriers such as poor tumor targeting, intratumoral spread, and virocentric immune responses. A deeper understanding of these barriers is essential to design more effective, which may be used alone or in combination with chemotherapy or immunotherapy 61, 62. In conclusion, adenoviruses are being actively explored and developed for the treatment of TNBC through various strategies, including combination therapies with CAR-T, ICIs, and genetic modifications to enhance their anti-tumor efficacy and overcome existing therapeutic challenges.

Herpes Simplex Virus (HSV) is being explored as a potential therapeutic agent in the treatment of TNBC. Oncolytic herpes simplex virus (oHSV) has shown great promise due to its ability to selectively infect and destroy cancer cells while sparing normal cells. This selective targeting is achieved through genetic modifications that enhance the virus's ability to replicate in tumor cells and express immunostimulatory molecules, thereby triggering an anti-tumor immune response 63, 64. Clinical trials are currently underway to evaluate the efficacy of HSV-based oncolytic therapies in various cancers, including TNBC. For instance, a phase II study is investigating the effects of adenovirus-mediated herpes simplex virus thymidine kinase expression combined with valacyclovir, stereotactic radiotherapy, and pembrolizumab in patients with TNBC 56.

The FDA has already approved T-VEC, an HSV-based oncolytic virus, for use in biological cancer treatment, highlighting the potential of HSV in cancer therapy 65. This approval underscores the safety and efficacy of HSV-based therapies, which are now being extended to other cancer types, including TNBC. Moreover, the combination of oncolytic viruses with other treatment methods such as chemotherapy, radiotherapy, and immunotherapy is being explored to enhance therapeutic outcomes. This approach aims to overcome the limitations of monotherapy and improve the overall efficacy of cancer treatments 66. In conclusion, HSV-based oncolytic virotherapy represents a promising avenue for the treatment of TNBC, with ongoing research and clinical trials aimed at optimizing its efficacy and safety 63, 65.

Reoviruses have shown promise in the treatment of TNBC, a subtype of breast cancer that is particularly challenging to treat due to its lack of hormone receptors and HER2 expression. The use of reoviruses as oncolytic agents leverages their ability to selectively replicate in and destroy cancer cells while sparing normal cells. Reoviruses are non-enveloped viruses with a segmented double-stranded RNA genome, and they exhibit an inherent preference to replicate in tumor cells, making them ideally suited for oncolytic virotherapies 67, 68. Their use as anti-cancer agents has been evaluated in several clinical trials, which revealed that intra-tumoral and systemic delivery of reoviruses are well tolerated 69.

In TNBC, reoviruses have been shown to increase cytotoxic T cell tumor infiltration and upregulate IFN-regulated gene expression and the PD-1/PD-L1 axis in tumors via an IFN-mediated mechanism. The addition of PD-1 blockade to reovirus treatment has enhanced systemic therapy in preclinical models. This suggests that combining reovirus therapy with immune checkpoint inhibitors could be a promising strategy for treating TNBC. Moreover, reoviruses can "hitchhike" on peripheral blood mononuclear cells and dendritic cells, thereby evading neutralizing antibodies of the host immune system. This cell carriage and targeted delivery, along with the triggering of the host immune response, contribute to the further advancement of reoviruses in cancer therapy 70.

The successful application of OVs, including reoviruses, in other cancers such as head and neck cancer and melanoma has promoted research into their use in TNBC 43. This research is crucial as TNBC is resistant to many conventional therapies, and new treatment approaches are urgently needed. In conclusion, reoviruses offer a promising new treatment approach for TNBC by leveraging their selective replication in tumor cells, ability to evade the host immune system, and potential to enhance the efficacy of immune checkpoint inhibitors 43, 69, 70.

Vaccinia virus (VV) has shown promise in the treatment of various cancers, including TNBC. VV possesses natural tumor tropism, which allows it to selectively infect and kill cancer cells while sparing normal cells. This characteristic has been successfully utilized in preclinical models of TNBC, as well as other cancers such as renal cell cancer, colorectal cancer, hepatocellular carcinoma, melanoma, and osteosarcoma 71. Clinical trials are currently underway to evaluate the efficacy of oncolytic viruses, including the GL-ONC1 Vaccinia oncolytic virus, in cancer therapy. These trials aim to determine the potential application of these viruses in treating various cancers, including TNBC 72.

The use of VV in cancer therapy is not limited to monotherapy. Combination strategies integrating VV with chemotherapy, radiotherapy, or immunotherapy have shown promise in improving therapeutic outcomes. These combination approaches aim to enhance the efficacy of VV and overcome the limitations of oncolytic virus monotherapy 66. VV has emerged as a potential candidate for cancer treatment due to its ability to infect a wide range of cancer cells. The safety and efficacy of different viral backbones, including VV, are being explored in clinical trials. Additionally, the potential combination of oncolytic VV with immunotherapy or traditional therapies is being investigated to enhance its therapeutic effects 73. Overall, VV represents a promising option for the treatment of TNBC, with ongoing research and clinical trials aimed at optimizing its use and improving patient outcomes.

Emerging oncolytic viruses (OVs) are showing promise in the treatment of TNBC, a subtype of breast cancer that is particularly challenging to treat due to the lack of targeted therapies. OVs are genetically engineered or naturally occurring viruses that selectively infect and kill cancer cells while sparing normal cells. They also stimulate anti-tumor immune responses, making them a multifaceted approach to cancer therapy. Several studies have highlighted the potential of OVs in TNBC therapy. For instance, the use of replication-competent viruses that selectively target and destroy cancer cells has rapidly evolved, with many innovative OVs entering clinical trials and demonstrating encouraging safety and efficacy 74. These viruses not only lyse tumor cells but also stimulate antitumor immune responses, which is crucial for the treatment of aggressive cancers like TNBC 75.

The development of OVs has led to a new class of cancer therapeutics, although their transition to the clinical setting has been slow due to the need for modifications to enhance their potency and selectivity 76. Despite these challenges, the multifunctional characteristics of OVs indicate good application prospects, especially when used in combination with other therapies such as radiotherapy, chemotherapy, and immunotherapy 77. Clinical trials have shown that OVs are well tolerated and safe for use in patients, displaying clinical activity in various advanced tumors, including some cases of breast cancer 41.

TNBC poses significant therapeutic challenges due to its aggressive nature, lack of hormone receptors, and absence of HER2 overexpression, which limit the efficacy of targeted therapies commonly used in other breast cancer subtypes.

## Specific oncolytic virus types holding promise in cancer

Currently, there are many types of OVs used in clinical cancer therapy. OVs can generally be divided into two categories: one is non-genetically edited viruses, which are mostly naturally occurring viruses that have not been genetically edited 78; the other is genetically modified viruses that have been edited to replicate specifically within tumor cells 78, 79. Non-genetically edited viruses are mainly represented by the M1 virus, reovirus, and Newcastle disease virus 28. The M1 virus has therapeutic effects on various tumors in animal experiments. By injecting the M1 virus into mouse models via the tail vein, it was found that its particles were mainly concentrated in tumor tissues, effectively inhibiting tumor growth. Moreover, co-administration with anti-cancer drugs such as casein kinase II inhibitors significantly enhances the anticancer activity of the M1 virus, with an increase of up to 3600-fold 80. From non-genetically edited to genetically edited, OVs encompass a wide variety of types and characteristics. Based on the different types of viral genome nucleotides, OV vectors can be divided into DNA virus vectors and RNA virus vectors 78. Among them, DNA viruses are mainly represented by HSV, AdV, VV, and parvovirus H1; RNA viruses are mainly represented by RV, coxsackie virus (CV), poliovirus (PV), measles virus (MV), NDV, and vesicular stomatitis virus (VSV) 81.

To date, there are not many types of oncolytic viruses studied for the treatment of breast cancer. The following introduces the main types and characteristics of oncolytic viruses that have been studied in TNBC and the progress of clinical research.

## Herpes simplex virus

HSV is an enveloped neurotropic double-stranded DNA virus with two serotypes, HSV-1 and HSV-2, being among the most extensively studied DNA viruses. HSV has a large genome (approximately 150 kb), and some genes are not essential for virus replication. This provides sufficient space for inserting exogenous functional genes without limiting the virus packaging efficiency, making it an attractive candidate vector in the field of OV therapy 82. HSV attaches to the host cell surface using its glycoproteins, particularly glycoprotein D (gD), which binds to cell surface receptors like nectin-1. This binding is followed by fusion of the viral envelope with the host cell membrane, mediated by other glycoproteins such as gB and the gH/gL complex 83. HSV can enter cells either through direct fusion at the plasma membrane or via endocytosis 84. Once inside, the capsid is transported to the nucleus along microtubules 83. The viral DNA is then injected into the nucleus through the nuclear pore complex, leading to the production of new virions 85. The first type (HSV-1) is commonly used for oncolytic virus therapy and has been widely used in cancer treatment. It is recognized as a potent activator of innate and adaptive immunity. Therapeutic forms of HSV-1 are created by modifying or deleting genes that are crucial for viral replication in normal cells but not in tumor cells, such as thymidine kinase (TK), ICP34.5 (required for viral replication in nerve cells), ICP6 (encoding the large subunit of HSV-1 ribonucleotide reductase), and ICP47^86^, ^87^. T-VEC is genetically created through the deletion of ICP34.5 and ICP47 and the insertion of the GM-CSF gene. The deletion of ICP34.5, which encodes the neurovirulence factor, stops virus replication in neurons but supports virus replication in tumor cells 88. Furthermore, in the place of ICP34.5, T-VEC contains two copies of GM-CSF, which promotes dendritic cell maturation. ICP47 encodes an inhibitor of antigen presentation that blocks MHC class I antigen presentation to CD8+ T cells 89. The deletion of ICP47 can promote immune responses against tumor cells 90.

In 2017, Phase I and Phase II clinical studies initiated the research on the combination of the oncolytic virus (talimogene laherparepvec, abbreviated as T-VEC) with standard chemotherapy for the treatment of early-stage triple-negative breast cancer. T-VEC is a modified herpes simplex virus type 1 that encodes granulocyte-macrophage colony-stimulating factor. The chemotherapy drugs used were anthracycline and taxane. The rationale for using the combination of T-VEC and chemotherapy is that the lymphocytes infiltrating the lesions of triple-negative breast tumors respond more significantly to this type of neoadjuvant therapy 91. Moreover, preclinical data also support the synergistic effect of oncolytic viruses and chemotherapy 92. However, the advancement of such clinical trials remains relatively slow.

In 2021, a Phase I clinical trial explored the maximum tolerated dose and safety of T-VEC combined with chemotherapy for neoadjuvant treatment of TNBC, showing that T-VEC had controllable toxicity and good safety 93. Subsequently, the research team conducted a Phase II clinical trial, enrolling 37 patients with stage III TNBC who received preoperative treatment with T-VEC combined with standard chemotherapy. The results showed that 45.9% of the patients achieved complete remission after treatment, and the two-year recurrence-free rate was approximately 89%, indicating that the combination of T-VEC and standard chemotherapy could enhance treatment response, with significant efficacy especially in high-risk early TNBC patients 53.

## Vaccinia virus

VV is an enveloped double-stranded DNA virus with a genome length of approximately 190kb, capable of carrying large segments of exogenous genes, and tends to infect metabolically active cells. VV binds to the host cell surface and enters primarily through endocytosis, a process facilitated by an entry fusion protein complex composed of eight viral proteins: A16, A21, A28, G3, G9, H2, J5, and L5. Subsequently, VV utilizes the host cell's cytoskeleton for transportation 94. Since the viral core contains enzymes required for the initiation of post-infection transcription, its replication and progeny assembly can occur in the endoplasmic reticulum (ER) within the cytoplasm 95. Currently, VV mainly enhances tumor selectivity by deleting thymidine kinase (TK), vaccinia growth factor (VGF), type I interferon-binding protein (B18R), etc^96^. Pexa-vec is an OV that activates systemic immune responses and inhibits tumor cells by expressing granulocyte-macrophage colony-stimulating factor (GM-CSF). It possesses two distinct infectious forms-intracellular mature virus (IMV) and extracellular enveloped virus (EEV). This characteristic allows for simultaneous intravenous and intratumoral injection, as well as the ability to evade neutralizing antibodies 97. In a recent study, OV was used as a vector for personalized neoantigen immunotherapy against triple-negative breast cancer to evaluate this therapeutic approach. This study used bioinformatic tools and cell-based assays to identify immunogenic neoantigens in samples from TNBC patients, human, and murine cell lines. The immunogenicity of the neoantigens was tested *in vitro* (human) and *ex vivo* (murine) T-cell assays. To evaluate the effectiveness of this approach, the authors used a preclinical model of TNBC, treating tumor-bearing mice with TNBC neoantigens and oncolytic VV, and assessed the impact on the induction of neoantigen-specific CD8+ T cells, tumor growth, and survival. The results successfully identified immunogenic neoantigens and generated neoantigen-specific CD8+ T cells capable of recognizing mutated genes expressed in human TNBC cell lines. The study demonstrated that by identifying immunogenic neoantigens and developing a delivery system through tumor-specific oncolytic VV, neoantigen vaccines could significantly induce neoantigen-specific CD8+ T cell responses with notable anti-tumor effects 98.

Moreover, the large coding potential of vaccinia virus vectors is a defining feature. However, limited regulatory switches are available to control viral replication and the timing and dosing of transgene expression to facilitate safe and effective vector delivery. Bell and his colleagues utilized principles of synthetic biology to adapt drug-controlled gene switches to control virally encoded transgene expression, including systems controlled by FDA-approved rapamycin and doxycycline. Using ribosome profiling to characterize viral promoter strength, they rationally designed fusions of operator elements from different drug-inducible systems with vaccinia virus promoters to produce synthetic promoters yielding robust inducible expression with undetectable baseline levels. They also generated chimeric synthetic promoters to provide additional regulatory layers for vaccinia virus-encoded synthetic transgene networks. These switches were applied to enable inducible expression of fusogenic proteins, dose-controlled delivery of toxic cytokines, and chemical regulation of vaccinia virus replication. The design of this engineered vaccinia virus-vectored oncolytic virus can precisely modulate transgene circuitry for targeted cancer therapy 99.

Another study employed single-chain antibodies (scFv) targeting the novel immune checkpoint molecule TIGIT to arm oncolytic vaccinia virus, constructing an engineered recombinant oncolytic vaccinia virus VV-scFv-TIGIT. This study found that intratumoral injection of VV-scFv-TIGIT significantly inhibited the growth of TNBC in mice and prolonged the survival time of tumor-bearing mice. Intraperitoneal injection of the recombinant oncolytic vaccinia virus cured 90% of mice with ascites tumors. Moreover, cured mice developed immunological memory that protected against re-challenge with the same type of tumor. Intratumoral or intraperitoneal injection of the recombinant oncolytic vaccinia virus could reshape the local tumor immune microenvironment, transforming 'cold' tumors with low immune cell infiltration into 'hot' tumors with high immune cell infiltration. Its combination with PD-1 or LAG-3 antibodies showed good synergistic effects. This study provides a promising candidate strategy for tumor immunotherapy 100.

## Reovirus

Reovirus is a non-enveloped virus with a double-stranded RNA genome, featuring an outer capsid and an inner core in its structure. Reovirus attaches to the host cell surface via junctional adhesion molecule-A (JAM-A), followed by entry into the host cell through clathrin-mediated endocytosis 101. After entry, reovirus is transported to early endosomes, where the outer capsid undergoes acid-dependent disassembly, and the transcriptionally active viral core is subsequently released 102. The transcription and translation events for progeny virus assembly occur within viral factories located in the cytoplasm 103. Reovirus naturally occurs in the mammalian respiratory and intestinal systems, exhibiting no apparent pathogenicity. However, it has a targeted lytic effect on cells activated by the rat sarcoma viral oncogene homologue (RAS) pathway. As over 80% of malignant gliomas have abnormalities in the RAS signaling pathway, malignant gliomas are often ideal candidates for RV therapy 104. Reolysin is an unmodified wild-type oncolytic reovirus. In 2017, Oncolytics Biotech announced results from a Phase II study at the American Association for Cancer Research (AACR) Annual Meeting, indicating that the combination of pelareorep and paclitaxel effectively extended overall survival (OS) in patients with metastatic breast cancer carrying p53 mutations, from 10.4 months to 17.4 months. Consequently, pelareorep received Fast Track designation from the FDA for the treatment of metastatic breast cancer 105. Clinical studies have demonstrated its significant effectiveness when used in combination with systemic anti-programmed cell death protein 1 (PD-1). In a mouse breast cancer model, intratumoral injection of reovirus increased PD-L1 expression on tumor cells, and combination reovirus/anti-PD-1 treatment significantly improved survival by reducing the number of regulatory T cells and enhancing tumor-specific cytotoxic T lymphocyte responses 106. Research has shown that neutralizing antibodies impede the oncolytic efficacy of reovirus but do not affect its immunotherapeutic capacity. Given the high seropositivity rate of reovirus in cancer patients, it is strongly recommended to use reovirus as part of T cell-based immunotherapeutic strategies 107. There is study demonstrates that the conjugation of doxorubicin with engineered reovirus facilitates the delivery of chemotherapy drugs to virus-targeted cells, enhancing the efficacy of killing TNBC cells 108. Other research indicated that when reovirus infection occurs alongside DNA-damaging agents, it amplifies the infection and eradication of TNBC cells. This implies that combining a genetically modified oncolytic reovirus with topoisomerase inhibitors could offer a robust treatment option for individuals suffering from TNBC 109.

## Adenovirus

Among oncolytic viruses, adenoviruses are the most widely used type. Adenoviruses (AdVs) belong to the family Adenoviridae and the genus mast adenovirus. These viruses have a double-stranded linear DNA genome, approximately 30-40 kb in size, and a non-enveloped icosahedral capsid 110. Adenovirus attaches to the host cell surface primarily via the coxsackie and adenovirus receptor (CAR) and enters the cell through clathrin-mediated endocytosis 111. Inside the cell, it is transported to early endosomes. The acidic environment of the endosome triggers partial disassembly of the viral capsid, allowing the viral DNA to escape into the cytoplasm and then be transported to the nucleus 112. Once in the nucleus, the viral DNA utilizes the host cell's machinery to begin transcription and replication 113. The replication cycle of adenoviruses can be divided into two stages; thus, the adenovirus genome is divided into early (E1-E4) and late (L1-L5) transcription units, the latter encoding structural proteins. The early region 1A (E1A) encodes five different proteins; early replication requires two major isoforms, 12S and 13S. The E1A 13S protein, composed of 289 amino acids, contains four conserved regions (CR1-CR4), which can trans-activate other early transcription units E1B, E2, E3, and E4, whereas E1A 12S lacks CR3^114^. Adenoviruses with deletions in the E1 gene are considered replication-defective and are used as shuttle vectors in gene therapy or vaccination. Replication-competent adenoviruses are known as conditional replication adenoviruses (CRAd) and are used clinically for oncolytic adenovirus therapy 115.

Recently, researchers have developed a variety of Oncolytic Adenoviruses (OAVs) equipped with multiple regulatory elements. This advancement has notably enhanced their specificity, effectiveness, and capacity to evade detection by the immune systems of patients, making them stand out as the most remarkable among all OVs 116, 117. Xie et al utilized synthetic biology principles to construct modular synthetic gene circuits that regulate oncolytic adenovirus replication selectively in tumor cells, thereby inducing specific tumor cell killing and stimulating anti-tumor immunity. This approach offers a novel solution for precise engineering and remodeling of oncolytic adenoviruses, enhancing the effectiveness and safety of oncolytic virus-targeted tumor immunotherapy. Moreover, Wei et al developed an oncolytic adenovirus encoding apolipoprotein A1 (AdV-ApoA1), which corrects aberrant cholesterol metabolism, reduces intracellular cholesterol levels, and leads to dephosphorylation of the transcription factor FOXO3, enabling its translocation into the nucleus. FOXO3 suppresses the transcription of the KRT14 gene by directly binding to its promoter region, thereby inhibiting the invasion and metastasis of TNBC cells 48.

## Measles virus

The measles virus (MV), classified under the genus Morbillivirus in the family Paramyxoviridae, is a single-stranded, negative-sense RNA virus. It comprises six major structural proteins, including the hemagglutinin (H) and fusion (F) glycoproteins. These glycoproteins are crucial for viral entry, as they facilitate the fusion of the viral envelope with the host cell membrane 118. MV primarily attaches to host cells through signaling lymphocyte activation molecules (SLAM, also known as CD150) and nectin-4 receptors. SLAM is predominantly expressed on immune cells, while nectin-4 is found on epithelial cells. The virus uses its hemagglutinin protein to bind to these receptors 119 Once internalized, MV replicates within the cytoplasm of the host cell.

As early as 2006, studies began to explore the potential of the measles virus (MV) in treating breast cancer. Research indicated that an MV strain engineered to produce carcinoembryonic antigen (MV-CEA) induced cell death in MDA-MB-231 breast cancer cells *in vitro*. Additionally, when tested *in vivo* using subcutaneous MDA-MB-231 xenografts, MV-CEA demonstrated significant therapeutic efficacy against triple-negative breast cancer (TNBC) both *in vitro* and *in vivo* 120. Further investigations revealed that these oncolytic viruses (OVs) enter cells via several receptors, including the signaling lymphocyte activation molecule (SLAM) expressed on various immune-associated cells, CD46 expressed on all nucleated cells, and poliovirus receptor-related protein 4 (PVRL4) 121 122. Building on this understanding, another research group engineered a modified MV, referred to as rMV-SLAM blind, which selectively used PVRL4 but not CD46 to infect TNBC cells, thereby avoiding SLAM-positive lymphoid cells. This rMV-SLAM blind variant significantly reduced the viability of breast cancer cell lines and exhibited greater oncolytic activity compared to wild-type MV, while also demonstrating a favorable safety profile 123 In 2020, the research team investigated the efficacy of rMV-SLAM blind against triple-negative breast cancer (TNBC). They found that the receptor for rMV-SLAM blind, Nectin-4, is expressed on the surface of 75% of TNBC cell lines. In their experiments, rMV-SLAM blind was used to infect Nectin-4 expressing TNBC cell lines, resulting in a significant reduction in the viability of approximately half of the breast cancer cell lines *in vitro*. Additionally, intratumoral injection of rMV-SLAM blind inhibited tumor growth in MDA-MB-468 and HCC70 cell xenografts 124. Collectively, these studies support the potential of MV as a promising candidate for TNBC treatment.

## Strategies for engineering oncolytic viruses and improve their targeting and safety

The modification of OVs refers to the alteration of their genomes using genetic engineering techniques to enhance selective oncolytic ability against tumor cells, improve therapeutic efficacy, or enhance their safety and stability. According to the primary purpose of their modification, the engineered oncolytic viruses are divided into the following categories: viruses that improve the targeting and safety, viruses that improve the efficacy of tumor therapy, and viruses that address limitations in conventional drug delivery routes. Gene deletion or silencing, by deleting or silencing specific regions of the viral genome, such as viral pathogenicity-related genes or essential replication genes, the virus's infectivity to normal cells can be reduced, while its ability to selectively kill tumor cells can be enhanced. For instance, Deletion viruses lack essential genes for replication in normal cells but do not affect their replication in tumor cells. For example, T-VEC (talimogene laherparepvec, Imlygic) is a type of HSV-1 virus with two deletions in its genome: RL-1 (expressing the neurotoxic factor ICP34.5, which can inhibit the host's antiviral immune response through the protein kinase (PKR) - interferon (IFN) pathway activated by double-stranded RNA) and α47 (expressing the early protein ICP47, which inhibits antigen presentation) 125. The absence of ICP34.5 expression prevents the virus from replicating in normal cells while not affecting its replication in tumor cells. In October 2015, the FDA approved the OVs drug T-VEC for the treatment of advanced melanoma in the United States. In 2016, T-VEC was approved for marketing in Europe and Canada, marking the maturity of OVs technology and the formal recognition of OVs in cancer treatment 126-128. Deletion of key viral replication genes allows the virus to replicate only in some tumor cells, with minimal presence or inability to proliferate in normal cells. For example, Pexa-Vec (JX-594), an oncolytic Vaccinia virus (VV) enhanced with GM-CSF, increases tumor selectivity by deleting the TK gene. Pexa-Vec has been evaluated in multiple phase I and II clinical trials in patients with colorectal cancer, hepatocellular carcinoma (HCC), and renal cell carcinoma.

Insertion of tumor- or tissue-specific promoters upstream of essential viral replication genes allows the virus to replicate only in tumors or specific tissues. Examples include the human telomerase reverse transcriptase (hTERT) promoter and the carcinoembryonic antigen (CEA) promoter 129, 130. Similarly, oncolytic adenovirus drugs like Onyx-015 achieve tumor selectivity by deleting specific viral genes. Onyx-015, for example, targets tumor cells by deleting the E1B-55 kDa gene, which typically inhibits virus replication by interacting with the tumor suppressor gene p53 in normal cells. Since many tumors harbor mutations in p53, the modified adenovirus can selectively replicate in tumor cells. Likewise, researchers have enhanced the specificity of oncolytic vaccinia virus by deleting genes like thymidine kinase (TK) from the viral genome. This modification prevents the virus from replicating in normal cells lacking TK, thereby enhancing its tumor-killing ability while minimizing off-target effects. Overall, these strategies aim to improve the targeting and safety of oncolytic viruses for cancer therapy, paving the way for more effective and selective treatments against various types of tumors, including breast cancer and its metastases.

Modifying the virus's structural proteins or envelope proteins to enhance its stability and safety *in vivo*, reduce its pathogenicity or immunogenicity, and decrease adverse reactions. Given that certain mutations commonly found in cancer, such as those in P53, RB1, PTEN, DCC, RAS, P16, and VHL genes, compromise cell antiviral capabilities, they frequently become prime targets for OV attacks 131. Although some natural virus strains exhibit a preference for tumor cells, their anticancer efficacy remains constrained, and controlling pathogenicity poses challenges. Genetic manipulations, encompassing gene element regulation and the incorporation of exogenous genes into engineered recombinant OVs, can enhance both safety and performance. Through these modification strategies, oncolytic viruses can possess stronger tumor specificity and therapeutic effects, thus becoming a powerful tool in cancer treatment.

In addition, inserting exogenous genes into the genome of oncolytic viruses can increase their anti-tumor effects or enhance immunogenicity or enhance the ability to regulate the tumor microenvironment. For instance, OVs locally expressing and releasing GM-CSF can promote the maturation and migration of DC cells, macrophages, and enhance T cell immune responses 132. Besides GM-CSF, other commonly used cytokines include IL-2, IL-12, IL-15, and more. Expression of chemokines: Chemokines, the largest subfamily of cytokines, mediate the migration of immune cells and lymphoid tissue development. Currently studied chemokines include CCL5 (which can increase the virus's survival time at the tumor site), CCL19 (controlling tumor growth, increasing DC, CD4+ T cell migration to the TME), CCL20, and CCL21^20^, ^55^, ^133^, ^134^. Studies on OVs expressing cluster of differentiation 40 ligand (CD40L) showed inhibition of tumor growth and upregulation of effector T cell proportions in the TME 135, 136. OVs expressing tumor necrosis factor superfamily member 4 (TNFSF4) promote the survival of effector T cells and memory T cells, maintaining their homeostasis while controlling and regulating T cell differentiation and functional expression 137. Furthermore, bispecific T cell engagers (BiTEs) consist of a single-chain variable fragment (scFv) arm that binds specifically to CD3 or other T cell activation receptors on the surface of T cells, and another scFv arm that bind to target antigens on tumor cell surfaces. Binding of both arms to their respective target antigens stimulates T cell activation, leading to apoptosis of target tumor cells. Due to the short half-life of BiTE molecules in serum, limited penetration into tumors, and dose-limiting toxicity, researchers have developed OVs encoding BiTE expression 138. The first OVs loaded with BiTE were oncolytic vaccinia viruses targeting tumor surface antigen ephrin A2 (EphA2-TEA-VV) 139. Studies found that EphA2-TEA-VV combined with peripheral blood mononuclear cells (PBMCs) significantly inhibited mouse lung cancer xenografts. Currently, several OVs based on this strategy are in preclinical and clinical research stages, such as ICOVIR-15K-cBiTE (targeting CD3/EGFR) and NG-641 (targeting CD3/FAP) 140.

## Methods to address limitations in conventional delivery routes

Previously, the primary method of delivery for OVs drugs was intratumoral administration, which maximized the distribution of OVs within the tumor 23. However, this approach has several drawbacks: (i) OVs cannot be successfully injected into tumors due to the dense and high-pressure nature of tumor tissue; (ii) Intratumoral injection is often inappropriate for patients with malignancies in deep organs; (iii) Compliance with intratumoral administration is poor, especially for those requiring continuous administration 141-143.

Compared with intratumoral injection, intravenous administration appears to be a better method for clinical application of OVs. Intravenous injection offers two significant advantages: it is highly convenient and feasible, and it possesses strong anti-metastasis and anti-recurrence capabilities 144. Despite these advantages, the actual outcomes of intravenous OV administration have been unsatisfactory. The primary challenges of systemic delivery are as follows: (1) Pre-existing and rapidly formed neutralizing antibodies in the systemic circulation significantly impede the delivery and reduce the therapeutic effect of OVs. (2) The concentration of OVs retained in the tumor area after systemic administration is lower than that achieved by intratumoral injection, and the risk of systemic side effects is higher 145. Overcoming these critical shortcomings would undoubtedly make intravenous oncolytic viruses the most promising method for tumor treatment. Therefore, researchers have adopted various modification strategies to enhance the effectiveness of intravenous OVs delivery, which can be categorized into two main approaches: cell carrier-based delivery and bioengineered material-based delivery.

Cell-based vectors are formed by wrapping OVs with cellular components such as cells or cell membranes to improve biocompatibility, circumvent antiviral immunity, or enhance anti-tumor immunity. Several promising candidates for safe and highly effective cell carrier systems, including mesenchymal stromal cells (MSCs), lymphocytes, white blood cells, and myeloid cells, have been explored for systemic OVT delivery. MSCs are the primary cell carriers used in this context. Their natural tumor tropism and ability to shield oncolytic viruses from the host immune system allow the viruses to effectively reach and infect tumor cells.

MSCs- encapsulated OVs provide an attractive systemic delivery option for the treatment of tumors. For instance, Stoff Khalili et al. used hMSCs loading conditionally replicating adenoviruses (CRAds) and treated MDA-MB-231-derived lung metastases by intravenous injection. The results showed that hMSCs-loaded CRAds could reduce the incidence of lung metastasis of breast cancer compared with direct injection of CRAds, most likely due to viral replication in the hMSCs 146. Similarly, Zhang et al investigated the therapeutic effect of injecting MSCs loaded with oncolytic adenovirus, a natural inhibitor of TGF-β signaling, into a mouse model of breast cancer with pulmonary metastasis. They demonstrated the therapeutic benefits of bone marrow mesenchymal stem cells promoting oncolytic adenovirus delivery and transmission in tumor tissue 147. In addition to metastatic breast cancer, metastatic melanoma (MM) is also a feared diagnosis among tumor patients due to its powerful proliferative and metastatic characteristics. The results of clinical statistical analysis indicate that more than 50% of patients with advanced melanoma will also develop MM-induced brain cancer 148. A study using mesenchymal stem cells armed with oncolytic herpes simplex virus (oHSV) to treat a mouse model of melanoma BMS found that intra-carotid administration of MSC-oHSV, rather than purified oHSV alone, was effective in tracking metastatic tumor lesions and significantly extended survival time in tumor-bearing mice. It is worth mentioning that the combination of MSC-oHSV and PD-L1 blocking increased IFN-γ-producing CD8+ tumor-infiltrating T lymphocytes and significantly extended the median survival of treated animals 149. A number of studies on the systematic delivery of mesenchymal stem cells as oncolytic virus vectors for tumor treatment have achieved surprising results, indicating the strong advantages of mesenchymal stem cells as oncolytic virus cell vectors T lymphocytes have also been explored as potential vectors for OVs. T cells can not only protect OVs from nab, but also load OVs and support subsequent viral amplification 150. Melzer et al. loaded vesicular stomatitis virus (VSV) onto CD8+ T cells, which had a better safety profile and more effective killing of myeloid leukemia tumor cells compared to naked VSV 151. The treatment of solid tumors with CRA-T presents significant challenges due to immunosuppressive TME 152. The use of OVs for oncolytic cell immunotherapy by infecting tumor cells may disrupt the TME 153. Zheng et al. used CAR T cells loaded with MYXV (Myxoma virus), a novel oncolytic virus, and found that this combination of drug delivery can effectively " lysing " tumors and even induce CAR-T antigen-negative tumor cell death via autosis (autophagy-dependent cell death) 154. Although the number of studies on T lymphocytes as vectors is limited, positive results suggest that they are potential vectors for bone marrow mesenchymal stem cells; However, further research is still needed.

In addition to the delivery of OVs by cell carriers, a variety of materials have also been used in the field of OVs system delivery, including bioactive polymer packaging, liposome packaging, nanohydrogels, etc. The use of bioactive polymers is an attractive delivery regimen that can enhance the protection of the host immune system and improve the efficacy of anti-cancer treatments. In 2021, Garofalo et al. designed a viral delivery platform to effectively treat hepatocytes by coating adenovirus Ad5/3-D24-ICOSL with a polygalactosyl-b-agmatyl diblock copolymer (Gal_32_-b-Agm_29_) 155. The study found that polymer-coated OVs may show significant improvements in infectivity, viral replication, lysis, and immunogenic cell death in tumor cells, with a high safety profile and an effective therapeutic effect. Similarly, in 2023 Liang et al proposed a surface engineering strategy to mask oncolytic herpes simplex virus (oHSV) with chains of galactose-polyethylene glycol (PEG) polymers to reduce host antiviral response and selectively target tumors by limiting circulating exposure during systemic administration. The results revealed that glycosylation-PEG modified oHSV not only did not affect the replication of oHSV, but also reduced the level of specific neutralizing antibodies and prevented tumor progression by reducing regulatory T cells and increasing the infiltration of activated CD8+T cells and NK cells 156. These findings demonstrate the potential of bioactive polymers for the systematic delivery of OVs. However, some studies have shown that the delivery efficiency of synthetic polymers as carriers is average and has high toxicity, which can lead to adverse side effects 157, 158. Therefore, subsequent studies should focus on the capture-release efficiency of biocompatible polymers and OVs, as well as polymers connected by dynamic covalent bonds or naturally derived polymers to overcome these shortcomings.

Liposome based viral encapsulation is another attractive strategy for systematic viral delivery. In 2017, Aoyama et al. used telomescan-specific oncolytic adenovirus expressing GFP (lipop-pts) encapsulated in liposomes to have a strong anti-tumor effect on HCT116 colon cancer cells both *in vitro* and *in vivo* 159. In 2019, Wang et al. used antitumor infection-resistant oncolytic alphavirus M1, encapsulated into liposomes (M-LPO), to kill and infect LoVo and Hep 3B cell lines, possibly due to the reduction of the virus's inherent immunogenicity and improved delivery efficiency 160. Another study synthesized cationic, 2-dioleyl-3-trimethylpropanolinium-folate liposomes (TAV255-Df) that encapsulated replication-deficient oncolytic adenovirus (OAd). The TAV255-Df liposomal encapsulation platform effectively circumvented the need to enter cells through coxackievirus and adenovirus receptors (CAR), and enhance the tumor killing effect of the virus. For example, in a CAR-deficient CT26 colon cancer mouse model, treatment of subcutaneous tumors by intratomatous injection of TAV255-Df with the ability to replicate resulted in 67% complete tumor remission, extended survival, and anti-cancer immunity when the mice were again attacked by cancer cells without further treatment 161. However, some unencapsulated OVs are occasionally observed because they are not loaded in liposomes and remain suspended. Therefore, purification of OVs from liposomal encapsulated OVs remains a challenge for this delivery platform.

Unlike conventional treatments such as chemotherapy and radiotherapy, OVs offer a more targeted approach, minimizing collateral damage 37. Furthermore, OVs play a crucial role in cancer immunotherapy by modulating the tumor microenvironment (TME). By altering the immune landscape within tumors, OVs can enhance the infiltration of immune cells and stimulate antitumor immune responses 21, 162, 163. This ability to harness the body's own immune system to fight cancer is a promising avenue in cancer treatment. Moreover, OVs can synergize with other anticancer therapies, such as immune checkpoint inhibitors or traditional chemotherapy, to enhance treatment outcomes 164, 165. By combining different treatment modalities, clinicians can potentially achieve better tumor control and improved patient outcomes. Overall, the development and utilization of OVs represent a promising frontier in cancer therapy, offering a targeted and immunomodulatory approach that holds great potential for improving patient outcomes 27, 166

## Current status of clinical research on oncolytic viruses

At present, the range of tumors treated with OV drugs mainly includes solid tumors, such as melanoma, hepatocellular carcinoma, colon cancer, breast cancer, glioblastoma, multiple myeloma, head and neck cancer, and malignant pleural mesothelioma 23. Although OVs can bring clinical benefits to patients with different types and stages of tumors, there are still challenges in treating solid tumors with OVs as a monotherapy. To overcome the limitations of OVs monotherapy, researchers have attempted combination therapies based on OVs, including combining them with radiotherapy, chemotherapy, immune checkpoint inhibitors (ICIs), and chimeric antigen receptor (CAR)-T cell therapies, showing significant synergistic effects 167.

OVs and radiotherapy complement each other mechanistically, showing synergistic effects. On one hand, radiotherapy causes DNA damage, and some OVs can sequester DNA damage response proteins, inhibiting DNA repair mechanisms, effectively acting as radiosensitizers. On the other hand, radiotherapy induces tumor cell apoptosis, releasing tumor-associated antigens (TAAs) and damage-associated molecular patterns (DAMPs), promoting OV replication and spread 168. In a phase I trial of the oncolytic HSV virus G207 combined with radiotherapy for recurrent malignant glioma, 6 out of 9 patients receiving the combination therapy showed synergistic activity 169.

Similar to radiotherapy, chemotherapy enhances the clinical benefits of OV therapy through various mechanisms, such as inhibiting antiviral immune responses, releasing TAAs, increasing tumor cell immunogenicity, and directly killing tumor cells and releasing viral particles 170. In a phase II trial for recurrent head and neck cancer, a modified oncolytic adenovirus ONYX-015 combined with cisplatin and 5-fluorouracil (5-FU) enhanced antitumor effects; the response rate for the combination therapy (OVs + cisplatin + 5-FU) was 63%, compared to only 15% for patients receiving ONYX-015 alone 171.

ICIs target checkpoint receptors or ligands such as programmed cell death receptor 1 (PD-1), programmed cell death ligand 1 (PD-L1), or cytotoxic T lymphocyte-associated antigen-4 (CTLA-4, also known as CD152), blocking tumor immune suppression signals and thereby restoring the body's antitumor immune response. Several ICIs have been approved by the US FDA, including CTLA-4 inhibitor (ipilimumab), PD-1 inhibitors (nivolumab, pembrolizumab, cemiplimab), and PD-L1 inhibitors (avelumab, durvalumab, and atezolizumab). As OVs can turn "cold" tumors into "hot" tumors by modulating the tumor microenvironment (TME), enhancing the antitumor activity of ICIs, the number of clinical trials investigating the combination of OVs and ICIs has increased in recent years. While TNBC as a subtype has a higher immunogenic potential, it is not universally "hot" across all cases. Some TNBC tumors might still evade the immune system or have areas within the tumor microenvironment that are "cold" or immunosuppressive. OVs could play a crucial role here by amplifying the immune response and overcoming these pockets of immune evasion, thereby converting partially "cold" or "immunologically silent" regions of the tumor into "hot" ones, enhancing the overall immune response and improving therapeutic outcomes. Combining ICIs and OVs can synergistically enhance immune responses and holds great therapeutic potential 137, 172, 173. A randomized, open-label phase II clinical trial (NCT01740297) for the treatment of advanced unresectable melanoma showed that the objective response rate (ORR) for patients in the T-VEC combined with CTLA-4 antibody ipilimumab group was 39% (38/98), compared to only 18% (8/100) in the ipilimumab monotherapy group. Among these, 13 patients in the combination group achieved complete response (CR), compared to 7 patients in the monotherapy group 166. In 2017, a multicenter phase Ib clinical trial evaluated the safety and efficacy of the combination of PD-1 antibody Keytruda and the oncolytic virus T-VEC. Results showed that the ORR in the combination therapy group was as high as 62%, with 33% achieving CR, which was much higher than the expected response rates (usually around 35% to 40%) for Keytruda or T-VEC monotherapy 174. In the near future, with more OVs and ICIs entering clinical development, this strategy is expected to bring more breakthroughs in cancer treatment. In preclinical studies, the oncolytic HSV1 HF10 combined with the EGFR tyrosine kinase inhibitor erlotinib demonstrated excellent anti-tumor activity in a pancreatic cancer mouse xenograft model 127.

Besides, CAR-T cell therapy involves genetically modifying T cells to express CAR, enabling CAR-T cells to recognize and kill tumor cells with specific antigens. Combining CAR-T cell therapy with genetically modified OVs can significantly induce CAR-T cell penetration into the TME and improve the therapeutic effect of CAR-T cells in solid tumors 175, 176.

## Regulatory challenges

Intratumoral injection poses a primary obstacle to the clinical application of OV, and increasing clinical data support intravenous administration, which allows OVs to infect all lesions widely and avoids the need for complex targeting devices. Studies have administered oncolytic vaccinia virus via intravenous injection to three melanoma patients and six colorectal cancer patients before surgical removal of metastatic lesions, demonstrating tolerable safety and detection of OVs in excised tumor specimens. However, the optimal dose for intravenous administration of OV remains unclear, as the virus is diluted in the peripheral circulation, making it difficult to predict the bioavailability at individual tumor sites. Furthermore, premature clearance of the virus by antibodies or complement in the systemic circulation may reduce the dose of OV reaching the lesions. Therefore, further research is needed on the pharmacokinetics of specific OV after entering circulation. New strategies such as nanocarriers or intracellular virus delivery may serve as protective measures for OVs during intravenous administration.

Although there are some OVs drugs have been approved globally, approval of other OVs is still subject to regulatory limitations. OVs have unique characteristics compared to other drugs. Firstly, OVs are replicating live viruses; secondly, OVs are mostly administered intratumorally. This renders many traditional approaches to determining clinical trial endpoints, pharmacokinetics, dosing, and regimens unsuitable for OV evaluation. Determining specific dosages poses significant challenges, as researchers must consider multiple factors, including the highest concentration achievable based on current Good Manufacturing Practice (cGMP) regulations, production capacity, viral immunogenicity, likelihood of pre-existing neutralizing virus antibodies, tumor histology, and other transgene expression influences. Other factors to consider include whether dosages should be adjusted based on tumor volume and the maximum safe dose determined by viral pathogenicity. The most suitable site for injection should also be chosen, considering the potential pathogenicity and immunogenicity of specific viruses. Given the unique pharmacokinetics of OV and the possibility of pseudo-progression, careful design of clinical trials to assess OV anti-tumor activity is necessary, including the selection of appropriate endpoints and controls.

## Future directions

Looking ahead, future research endeavors should prioritize the development of strategies to mitigate the clearance of intravenously administered OVs, ensuring their effective delivery to tumors and subsequent replication. Overcoming this challenge is crucial as the antiviral immune response triggered by OVs can impede the efficacy of oncolytic virotherapy. Additionally, there is a need to explore the optimal timing for OV administration. Given the absence of viable alternatives to surgical interventions, determining whether oncolytic treatment is most efficacious pre- or post-surgery is imperative. This consideration is particularly significant since OV replication relies on viable tumor cells, which may be compromised post-tumor resection, potentially reducing therapeutic efficacy.

Drawing from previous clinical insights, combination therapy emerges as the preferred treatment modality. Therefore, additional data elucidating combination therapies tailored specifically for TNBC are warranted. Moreover, deeper elucidation of the underlying mechanisms driving the synergistic effects of combining OVs with radiotherapy or chemotherapy is imperative. It's noteworthy that combined therapies need not be restricted to just two modalities. Hence, evaluating the efficacy of additional combination approaches is essential (**Table 2**).

In addition, the utilization of tumor-specific promoters represents a significant advancement in enhancing the targeting of oncolytic viruses (OVs) to tumors. By incorporating these promoters, researchers can regulate the expression of key viral genes such as E1A, which is critical for viral replication. Numerous tumor-specific promoters have been explored, including human telomerase reverse transcriptase promoter (hTERT), hypoxia response element (HRE) promoter, prostate-specific antigen promoter (PSA), alpha-fetoprotein promoter (AFP), alpha-lactalbumin promoter (ALA), and mucin 1 promoter (DF3/MUC1). These promoters enable selective replication of oncolytic adenoviruses in tumor cells, thereby enhancing the specificity of oncolytic virus therapy.

In addition to tumor-specific promoters, researchers have devised innovative gene circuits incorporating microRNAs (miRNAs) to enhance the specificity and efficacy of oncolytic virus therapy. miRNAs are endogenous non-coding RNAs that regulate gene expression post-transcriptionally and play crucial roles in various physiological and pathological processes, including cancer progression. Aberrant expression of miRNAs in cancer cells presents an opportunity for therapeutic intervention.

One approach involves using OVs as gene delivery vehicles to introduce tumor-suppressive miRNAs into cancer cells. These miRNAs can inhibit oncogenic pathways or promote apoptosis, effectively suppressing tumor growth. For example, oncolytic adenoviruses carrying tumor suppressor miRNA143 have demonstrated anti-tumor effects by inducing apoptosis and reducing the expression of oncogenes in various cancer models 177. Overall, these innovative strategies hold promise for enhancing the specificity, efficacy, and safety of oncolytic virus therapy in cancer treatment. By exploiting tumor-specific promoters, miRNAs, and optogenetics, researchers are advancing the field towards more precise and targeted approaches for combating cancer.

In addition, future research should also focus on elucidating the molecular mechanisms underlying the interaction between oncolytic viruses and the TNBC tumor microenvironment. For instance, further studies are needed to explore the role of tumor-associated macrophages (TAMs) and myeloid-derived suppressor cells (MDSCs) in mediating resistance to oncolytic virotherapy. Additionally, the development of novel viral vectors with enhanced tumor tropism and reduced immunogenicity remains a critical area of investigation. By leveraging advances in synthetic biology and gene editing technologies, researchers can design next-generation oncolytic viruses that are more effective in targeting TNBC and overcoming the limitations of current therapies.

## Conclusion

OVs represent promising candidates for TNBC treatment due to their ability to replicate within tumor cells, resulting in direct lysis, destruction of tumor vasculature, and activation of innate and adaptive immune responses, leading to antitumor effects. They can also convert 'cold' tumors into 'hot' ones and serve as vectors for delivering target genes and expressing antitumor factors within tumor cells. These features offer advantages over conventional modalities like radiotherapy, chemotherapy, and endocrine therapy. Additionally, OVs' selectivity for tumor cells minimizes adverse events in normal cells and reduces the development of drug resistance. Furthermore, their ability to activate the immune system upon replication sustains a long-term antitumor effect. Therefore, investigating the feasibility of OVs for TNBC treatment is crucial for exploring new research avenues and establishing a novel clinical treatment platform.

## Figures and Tables

**Table 1:** Clinical trials with oncolytic viruses involving breast cancer.

Virus	Modification	Combination	Phase	RoA	Country	References	
Talimogene laherparepvec (HSV-1)	γ34.5-deleted; Armed with GM-CSF	Paclitaxel	I&II	I.T	USA	NCT02779855	
Talimogene laherparepvec (HSV-1)	γ34.5-deleted; Armed with GM-CSF	Ipilimumab;Nivolumab	I	I.T	USA	NCT04185311	
HF10 (HSV-1)	Spontaneously mutated	None	I	I.T	Japan	NCT01017185	
Pelareorep (Reovirus)	Naturally oncolytic	Paclitaxel;Avelumab	II	I.V	USA	NCT04215146	
Pelareorep (Reovirus)	Naturally oncolytic	Letrozole; Atezolizumab; Trastuzumab	I	I.V	Spain	NCT04102618	
PVSRIPO (Poliovirus)	Insertion of heterologous IRES from human rhinovirus type 2	None	I	I.T	USA	NCT03564782	
Pexa-Vec (Vaccinia virus)	Deleted of J2R; encodes GM-CSF	Ipilimumab	I	I.T	France	NCT02977156	
Pexa-Vec (Vaccinia virus)	Deleted of J2R; encodes GM-CSF	Cyclophosphamide	I&II	I.V	France	NCT02630368	
TBio-6517 (Vaccinia virus)	25 kb deletion from the virus genome	Pembrolizumab	I&II	I.T	USA	NCT04301011	
MV-NIS (Measles virus)	Encoding thyroidal sodium iodide symporter (NIS)	None	I	I.T	USA	NCT01846091	
Ad-RTS-hIL-12 (Adenovirus)	Nonreplicating adenoviral vector for interleukin-12; with veledimex	veledimex	I&II	I.T	USA	NCT02423902	
Ad5CMV-p53 (Adenovirus)	Adenovirus-mediated p53	None	I	I.T	USA	NCT00004038	
Adenovirus	Encodes HSV-TK	Valacyclovir; Pembrolizumab; radiation therapy	II	I.T	USA	NCT03004183	
CAdVEC (Adenovirus)	HER2-specific CAR-T cells	HER2-specific CAR-T cells	I	I.T	USA	NCT03740256	

RoA route of administration, i.t. intratumoral, i.v. intravenous

**Table 2:** Limitations and future directions for oncolytic virus therapy in TNBC.

Limitations	Rationale	Future Direction
Tumor microenvironment heterogeneity: Immunosuppressive cells and extracellular matrix barriers in the tumor microenvironment limit OV spread and efficacy; Abnormal tumor vasculature affects efficient delivery of OVs, preventing the virus from penetrating deeper tumor regions.	The tumor microenvironment in breast cancer plays a critical role in resistance to therapies, including oncolytic viruses. Overcoming these barriers could enhance OV efficacy and allow for better tumor penetration and destruction.	Further optimization of viral design and modification to improve penetration and functionality within the complex microenvironment; Combining OVs with immune modulators or agents that alter the extracellular matrix and vasculature to improve OV access to deeper tumor areas.
Host antiviral Immune Response: The innate immune system quickly recognizes and neutralizes OVs, reducing their lifespan and efficacy; Neutralization by antibodies or the complement system, particularly after repeated dosing, further limits the therapeutic potential of OVs.	The immune system's natural response to clear viruses hinders OVs from exerting a sustained effect. Therefore, combining OVs with immune-regulating therapies or chemotherapy may boost their effectiveness by weakening the host's antiviral defenses and enhancing the tumor-specific immune response.	Explore combination therapies with chemotherapy, immunotherapy, or immune checkpoint inhibitors to prolong OV activity and enhance antitumor responses; Develop strategies to modulate cytokine profiles and stimulate adaptive immune cells to sustain the OV's presence and efficacy in the body.
Delivering the OVs: Difficulty in delivering OVs to distant metastatic sites, particularly through intra-tumoral administration; Challenges with effective systemic delivery while maintaining tumor specificity.	Metastatic breast cancer is often systemic, requiring an OV delivery method that can reach distant tumors without losing functionality or specificity. Intra-tumoral delivery, while effective in localized settings, is not feasible for widespread metastatic disease.	Improve delivery vectors, such as nanoparticle-based carriers or other innovative platforms, that can target tumors systemically while bypassing barriers; Explore more effective systemic administration methods that preserve tumor-specific targeting.
Precision in OV targeting: Current OVs may affect normal cells due to incomplete selectivity, potentially leading to off-target effects and collateral damage; Enhancing tumor cell specificity without compromising the virus's ability to effectively kill cancer cells remains a significant challenge.	The precision of OV targeting is vital for minimizing damage to healthy tissue while maximizing cancer cell lysis. Genetically engineered OVs with improved selectivity will reduce adverse effects and improve patient outcomes.	Develop more precise targeting mechanisms through nanotechnology or genetically engineered viruses with enhanced selectivity and penetration into the tumor interior; Focus on enhancing viral gene editing techniques to improve the specificity of tumor cell targeting.
Safety and toxicity of treatment: High doses of OVs can trigger significant immune responses and systemic inflammation, leading to adverse effects; Replication of OVs in the host may cause immune toxicity, which can reduce patient tolerance to treatment.	While OVs are promising, their safety profile remains a major concern, especially when delivered at therapeutic doses. Personalized viral platforms and predictive biomarkers could help in minimizing toxicity and tailoring treatments to individual patients.	Develop personalized viral platforms to match the most suitable OV with each patient, based on their tumor and immune characteristics; Identify new biomarkers to predict patient response to OV therapy, allowing for more personalized and safer treatment protocols.
